# How Does Social Support Affect the Retention Willingness of Cross-Border E-Commerce Sellers?

**DOI:** 10.3389/fpsyg.2021.797035

**Published:** 2022-01-20

**Authors:** Huiyun Shen, Jie Yu, Hua Zhang, Jin Gou, Xiangqian Zhang

**Affiliations:** ^1^School of Business Administration, Huaqiao University, Quanzhou, China; ^2^College of Computer Science and Technology, Huaqiao University, Xiamen, China; ^3^School of Humanities, Shanghai Institute of Technology, Shanghai, China

**Keywords:** cross-border e-commerce, social support, perceived benefits, perceived usefulness, sellers’ willingness to retain, S-O-R theory

## Abstract

E-commerce research usually focuses more on how to protect consumers’ rights and increase their purchase intention from the perspective of consumers. However, we still lack understanding of e-commerce sellers, especially cross-border e-commerce sellers. Based on the stimulus-body-response theory, this paper built a moderated mediation model to test the relationships among social support, perceived benefits, perceived usefulness and sellers’ willingness to retain. The results show that social support has a positive impact on perceived benefits and sellers’ willingness to retain; perceived benefits play a partial intermediary role between social support and sellers’ willingness to retain; and perceived usefulness moderates these mediating effects. The research results further expand the perspective of e-commerce research and reveal the mechanism and boundary conditions of the influence of social support on the retention willingness of cross-border e-commerce sellers.

## Introduction

Online shopping has important economic potential and its popularity is gradually increasing. While online shopping brings convenience to buyers, it also brings benefits to sellers. However, the complexity of online shopping, especially cross-border shopping, has led to opportunistic behavior of buyers and sellers to a certain extent. It is worth noting that, by reviewing the previous literature, we found that most of the previous studies were from the perspective of consumers. They assume that consumers are the weaker party in online shopping, discuss how to protect consumers’ rights (Wang et al., 2021) and increase consumers’ willingness to buy (Escandon-Barbosa and Rialp-Criado, 2019; Liu et al., 2020; Guitart and Stremersch, 2021). They believe that opportunistic behavior of sellers seems particularly common in these computer-mediated trading environments (Hong, 2015; Kim and Koo, 2016). For instance, buyers in e-commerce usually conduct transactions with most unknown sellers. These sellers can easily create and change their identities and are basically unable to meet with buyers (Meents and Verhagen, 2018). As a result, some sellers deliberately provide false advertisements, sell unreliable products and other deceptive behaviors (Román, 2010), which will cause consumers not being able to obtain the authenticity information of the products.

However, considering the problem of widespread buyer opportunistic behavior such as chargeback fraud that is common in cross-border e-commerce transactions, this assumption is not always valid (Guo et al., 2018). Refund fraud refers to the behavior of buyers requesting the seller to refund the purchase price through some excuses, such as “unauthorized transaction,” and when the seller has returned the purchase price without returning the purchased goods to the seller, in order to obtain “free” goods (Khan, 2015). Besides, many third-party trading platforms have introduced policies that buyers can return goods when they are dissatisfied with goods within 180 days, which further strengthens refund fraud (Clemons, 2007), harms the seller’s interests and survival. Cross border transactions and delivery face greater complexity and high risks than traditional offline markets or domestic electronic markets. Compared with domestic transactions, the unique high transportation costs of cross-border transactions, the asymmetry of information between buyers and sellers, and the differences in laws, languages and cultures of various countries all determine that multinational e-commerce has a high degree of uncertainty (Gessner and Snodgrass, 2015). Guo et al. (2018) found through a study of 443 cross-border e-commerce sellers that in cross-border e-commerce transactions, sellers are full of uncertain about the success of the transaction like buyers, and once it happens in transaction disputes, sellers suffer far more losses than buyers. In addition, because some regulations of the third-party trading platform are too partial to consumers, placing sellers in a disadvantageous position, which affects sellers’ benefits and transaction willingness to some extent (Guo et al., 2018). This has also made many small and medium-sized enterprises “failed” after getting involved in cross-border e-commerce. According to statistics, in 2017, 391905 new sellers settled on Amazon’s US site, less than 30% of them sold goods, and the retention proportion of sellers was less than 10% (Li, 2018). Besides, the recent Sino US trade friction and the continuous spread of covid-19 have led to the rise of international logistics costs, which have brought great challenges to cross-border e-commerce sellers. In addition, since May 2021, the Amazon platform has closed more than 50,000 Chinese cross-border e-commerce sellers, which has caused an estimated industry loss of more than 100 billion yuan (Ma, 2021). All of these shows that China’s cross-border e-commerce is facing challenges from many aspects. Thus, sellers’ “difficulty to retention” has become a major problem restricting the development of cross-border e-commerce.

From the perspective of sellers, there are relatively few papers study on factors affecting sellers’ retention intentions. Most of existing research focuses on the study of consumer behavioral intentions, emphasizing how to protect consumers from sellers’ opportunistic behavior (Wang et al., 2021) and increase consumers’ willingness to buy (Escandon-Barbosa and Rialp-Criado, 2019; Liu et al., 2020; Guitart and Stremersch, 2021). Although previous research has enriched our understanding of cross-border e-commerce buyers and provided useful suggestions for improving consumers’ purchasing intentions. But as the other side of the transaction, cross-border e-commerce sellers play an important role in cross-border e-commerce transactions (Guo et al., 2018) and make a huge contribution to China’s foreign trade exports. It is critical for the development of China’s foreign trade to solve the problem of “difficulty to retention” of cross-border e-commerce sellers. Therefore, this research aims to meet the above-mentioned research gap by focusing on the retention willingness of sellers in the context of cross-border e-commerce. Based on stimulus-body-response (S-O-R) theory, we proposed a conceptual model. This model divides the social support obtained by cross-border e-commerce sellers into two dimensions: information support and emotional support. We use perceived benefits as an intermediary variable, and sellers’ perceived usefulness of third-party trading platforms as a moderating variable to test the influence mechanism and boundary conditions between social support and sellers’ willingness to retain.

This article has contributed to the literature related to cross-border e-commerce in the following aspects. Firstly, different from the existing literature that focuses on the protection of buyers’ rights (Wang et al., 2021) and the promotion of purchase intentions (Escandon-Barbosa and Rialp-Criado, 2019; Liu et al., 2020; Guitart and Stremersch, 2021). This article reveals the mechanism that can enhance sellers’ willingness to retain from the perspective of sellers. It has played a certain role in promoting the research of seller behavior in the cross-border e-commerce environment. The findings of this study are an important supplement to the existing literature on improving buyers’ purchase intentions. It not only enriches the research content of the existing cross-border e-commerce literature, but also expands the perspective of cross-border e-commerce research. Secondly, this paper found that social support and perceived benefits have significant influence on sellers’ retention intention, which challenges the dominant assumptions of the existing literature. The success of cross-border transactions mainly depends on buyers’ purchase intention, because buyers are affected by sellers’ opportunistic behavior. As an alternative, this research shows that sellers are also vulnerable to buyer fraud, trade frictions and other external factors. The social support and perceived benefits they receive influence the seller’s willingness to retain. Finally, this study provides strong evidence that third-party trading platforms need to strengthen the protection of sellers’ rights in the context of cross-border e-commerce. Specifically, as the main place for cross-border transactions, third-party online transaction platforms are critical to the development of cross-border e-commerce. However, existing research focuses on the protection of consumers’ rights (Wang et al., 2021) and the enhancement of consumers’ willingness to buy (Escandon-Barbosa and Rialp-Criado, 2019; Liu et al., 2020; Guitart and Stremersch, 2021). This article explores the moderating effect of the platform’s perceived usefulness on the relationship between the seller’s perceived benefits and retention intention, and provides a new perspective for the research on the platform’s perceived usefulness. Together, these studies provide a more complete portrait of how third-party trading platforms can play an important role in the sustainable development of cross-border e-commerce.

## Theoretical Basis and Research Model

### Theoretical Basis

The S-O-R theory originated from environmental psychology (Mehrabian and Russell, 1974). It points out that when an individual is stimulated, its internal state will change, which eventually leads to a certain behavior. With the development of e-commerce, many scholars use the S-O-R theory to explain user behavior. In existing studies, stimulus factors mainly include social factors (Huang, 2016), technical factors (Zhang et al., 2014), etc.; organic factors mainly include attitude (Chang et al., 2015) and trust (Tuncer, 2021), etc.; reactive factors mainly include willingness to share (Chang et al., 2015) and purchase intention (Liu et al., 2016) etc. It can be seen that the S-O-R theory has a certain explanatory power for people’s decision-making behavior. Thus, this research is based on the S-O-R theory to construct a theoretical model that affects the retention willingness of cross-border e-commerce sellers. According to S-O-R theory, social support is the stimulus element, the perceived benefit is the organic element, and the seller’s willingness to retain is the response element.

**FIGURE 1:**
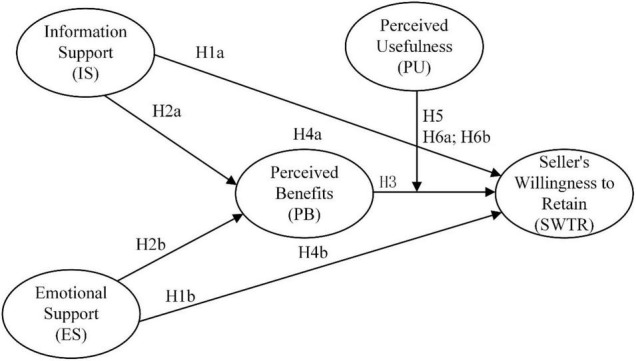
Research model. H4a-Indirect influence of IS on SWTR; H4b-Indirect influence of ES on SWTR; H6a-the conditional indirect relationship of IS with SWTR (through PB); H6b-the conditional indirect relationship of ES with SETR (through PB).

Specifically, in terms of stimulus factors, the research on social support involves multiple fields, and there is no unified definition in each field. However, it is a consensus that social support is a multi-dimensional concept. Schaefer et al. (1981) proposed that social support can be divided into information support, emotional support and tangible support in traditional social relations. More specifically, tangible support includes providing loans or material assistance. Information support refers to providing information, suggestions and timely feedback that can help solve problems. Emotional support refers to providing empathy, understanding or caring and other emotional support. With the development of information technology, traditional offline social relationships have been changed. People’s interactions are more online, which is virtual in nature. Therefore, compared with information and emotional support, there is little tangible support. Based on the above discussion, this study adopts the classification of social support by Liang et al. (2011) and Hajli (2014a), dividing social support into two dimensions: emotional support and information support. In terms of organic elements, the definition of perceived benefits is proposed to adopt the research of Molla and Heeks (2007) on the analysis of e-commerce advantage factors. It means that sellers feel the benefits of engaging in e-commerce, which mainly include reducing costs, increasing revenue and improving company competitiveness. In addition, we combine the definitions of Guo et al. (2018) on seller’s intention to trade and Liang et al. (2011) on continuance intention, and define seller’s retention intention as the seller’s willingness to persist in cross-border e-commerce when they have the opportunity to choose in the future. In addition, from the perspective of sellers, one of the main reasons for sellers to choose cross-border transactions is to attract foreign consumers, expand product markets and improve sales performance. This needs to rely on the “referral traffic” function of third-party platforms to achieve. Considering that the success of cross-border e-commerce requires the guarantee of third-party trading platforms, we incorporate sellers’ perception of the usefulness of third-party platforms into our research framework, and the research model of the entire research is shown in Figure 1.

### Research Model

In view of the importance of cross-border e-commerce to economic development and the complexity of cross-border transactions, many scholars have explored how to promote the development of cross-border e-commerce. However, most of the previous studies have focused on cross-border e-commerce buyers and neglected to sellers. As shown in the review of previous relevant literature in Table 1, previous research focuses on improving the benefits and (continuous) purchase intentions of cross-border e-commerce buyers. While these studies have greatly increased our understanding of cross-border e-commerce. However, considering the importance of sellers, the difficulty to retention and the risks of engaging in cross-border transactions. It is crucial to explore how to improve sellers’ interests and willingness to retain, in order to promote the healthy and sustainable development of cross-border e-commerce. Thus, we explore the impact of social support on the willingness to retain from seller’s perspective, drawing on online shopping-related research and SOR theory.

**TABLE 1:** Previous studies related to our research.

Author	Theme	Research object	Variable	Findings
Lin et al., 2018	Social support and consumer engagement in social commerce	Consumer	Social support; Social shopping intention; Social sharing intention	The social support influence user behaviors on their social shopping intention and social sharing intention.
Zhu et al., 2016	Social support on customer satisfaction and citizenship behavior in online brand communities	Consumer	Social support; Consumer satisfaction; Consumer citizenship behavior	Social support significantly affects the customer citizenship behavior through customer satisfaction in online brand communities.
Wang et al., 2020	Social support promotes consumers’ engagement in the social commerce community	Consumer	Social support; consumer engagement	Social supportive significantly affects the consumers’ engagement in the community through consumer involvement.
Shah et al., 2019	Perceived social support and perceived benefit	SNSs users	Perceived social support; Perceived benefit	Perceived social support is positive significantly associated with perceived benefits.
Park et al., 2019	Perceived benefit and consumers’ M-payment service adoption	Consumer	Perceived benefit; Intention to use	The perception of benefits in using the m-payment positively affects consumers’ intention to use M-payment.
Zhao et al., 2020	Consumers’ perceived benefit and their purchase intention.	Consumer	Perceived benefit; Purchase intention	Consumers’ perceived benefit is positively related to their purchase intention.
Payakkapong et al., 2017	Perceived benefit and sellers’ behavior in e-commerce	Sellers in E-commerce	Perceived benefit; Intention to use e-commerce	Perceived benefits had direct effects on the intention to use e-commerce.
Sun, 2010	Sellers’ trust and continued use of online marketplaces	Online Sellers	Sellers’ trust; Sellers’ retention of online marketplaces	A seller’s perceived usefulness of using an online marketplace for selling positively influences his/her retention of that marketplace.
Lee et al., 2019	Perceived usefulness and platform-based mobile payment service	Consumer	Perceived usefulness; Intention to use	Perceived usefulness positively affects user attitudes toward mobile payment services.
Abbes et al., 2020	Perceived usefulness and online collaborative redistribution platforms	Consumer	Perceived usefulness; platform behavioral intentions	Perceived usefulness of the platform has a direct positive influence on platform behavioral intentions.
Kwak et al., 2020	Perceived usefulness and green logistics platforms	Consumer	Perceived usefulness; Intention to use	The perceived usefulness for users increases the intention to use the green logistics platform.

In a virtual environment, feeling social support from other users will encourage people to be more open to business strategies (Yahia et al., 2018). In a situation with high-level social support, trust can be easily formed and is related to positive results. While a low level of social support can lead to despair and emotional problems (Shah et al., 2019). Past research has revealed that the social support consumers receive from friends, social media and other channels positively affects their satisfaction (Zhu et al., 2016), participation (Wang et al., 2020), citizenship behavior (Zhu et al., 2016), and purchase intention (Lin et al., 2018) in social commerce. Sellers, as another main body of the e-commerce market, also feel the social support from other practitioners. According to the S-O-R theory, when the seller perceives the stimulation of social support, the psychological perception will change and then take action. Because social support helps to increase the individual’s perceived benefits and reduce the individual’s perceived risk (Shah et al., 2019). Social support is considered a key indicator of participation and positive behavior (Hu et al., 2019). Therefore, the perceived social support of sellers can help increase their perceived benefits of engaging in cross-border e-commerce, which is consistent with the S-O path of the SOR theory. At the same time, social support will directly affect individual behavioral decisions (Lin et al., 2018; Hu et al., 2019). Therefore, the social support perceived can also help increase sellers’ willingness to engage in cross-border e-commerce.

In addition, many studies have shown that perception affects individual behavior (Dijksterhuis and van Knippenberg, 1998). For instance, when consumers perceive the benefits from mobile payment and online shopping, their use of mobile payment and purchase willingness will increase (Park et al., 2019; Zhao et al., 2020). Similarly, when sellers perceive the benefits of using e-commerce, their intention to use e-commerce will increase (Payakkapong et al., 2017), which is consistent with the 0-R path of SOR theory.

What’s more, from the perspective of sellers, they need to obtain adequate protection from third-party trading platforms for continued operation. Existing studies have extensively referenced the technology acceptance model to study user acceptance of e-commerce. But most of them studied the acceptance of mobile payment platforms (Lee et al., 2019), collaborative redistribution platform (Abbes et al., 2020), green logistics platforms (Kwak et al., 2020), and other e-commerce platforms from the perspective of consumers. Some scholars explained that sellers’ perceived usefulness of the e-commerce market can also significantly increase their retention rate (Sun, 2010). Cross-border e-commerce sellers often choose to stay on a third-party platform if they can perceive that using that platform will provide sufficient protection for their legal rights. The higher the perceived usefulness of the platform, the more cross-border e-commerce sellers believe that they can turn the perceived benefits into real benefits, thereby further enhancing the relationship between perceived benefits and sellers’ willingness to retain. And the higher level of perceived usefulness will also affect the indirect effect of social support on sellers’ willingness to retain through perceived benefits. This is because at a higher level of perceived usefulness, sellers are more convinced that information from social support is reliable and valuable, thereby increasing their perception of benefits and increasing their willingness to retain. Based on the above analysis, the conceptual model of this research is shown in Figure 1.

## Research Hypothesis

### Social Support and Sellers’ Willingness to Retain

Social support will have a positive impact on sellers’ willingness to retain. Social support is the experience of an individual being cared for and helped in a group (Cobb, 1976). It arises from the interaction between individuals in the group and represents the available social resources that a person perceives. These social resources make a person think that he is loved, respected and cared for. Existing literature has confirmed that social support will affect consumers’ purchase intention (Li, 2019), and even stimulate consumers’ impulse consumption (Hu et al., 2019). Similarly, this study believes that the amount of social support will affect the willingness of cross-border e-commerce sellers to retain. First, when there is more information support in a group, group members will naturally share business information and suggestions as an extension of their sharing of other support information (Liang and Turban, 2011). Second, if group members believe that other members have been providing helpful information, then obtaining or sharing valuable information with others will be considered a mandatory behavior (Crocker and Canevello, 2008). Finally, when individuals receive support from others, people often believe that they are obligated to give others similar support in return (Cheshire, 2007), and frequent sharing of useful information can enhance the trust and friendship between members (Liang and Turban, 2011). The frequent exchange of information among members of the seller group puts sellers in a mutually helpful situation. This mutually help process will generate information related to product marketing or sales. The existence of friendship and trust makes people feel that information is trustworthy and valuable for conducting cross-border transactions. Valuable information can help sellers reduce the uncertainty of transactions, avoid some speculative behaviors similar to refund fraud by buyer, and increase the probability of successful transactions. Besides, past successful transaction experience will enhance sellers’ confidence in cross-border transactions, thereby enhancing the willingness of sellers to continue cross-border business activities. Similarly, when a person gets more emotional support, it shows that he gets more care, trust, respect and understanding from others, which will make him feel that he is important, concerned and valuable (Chiang and Huang, 2015). This will provide sellers with a sense of belonging, thereby enhancing the willingness of sellers to retain. Thus, we put forward the following hypothesis.

H1a: Information support is positively associated with sellers’ willingness to retain.
H1b: Emotional support is positively associated with sellers’ willingness to retain.

### Social Support and Perceived Benefits

Perceived benefits are relative to perceived risks. It is an individual’s perceptual utility to provide things, which is generated based on the comprehensive judgment of the individual after weighing the benefits and costs (Woodruff, 1997). For the cross-border e-commerce seller in this study, the perceived benefit refers to the benefits that sellers feel that cross-border e-commerce brings to the enterprise. It should include the reduction of transaction costs, the improvement of company performance, and the improvement of competitiveness. However, in developing countries, due to the backwardness of technology and facilities, the cost for enterprises to obtain information is higher than the global average. The benefits of e-commerce are generally limited to the improvement of inter-organizational communication. Most enterprises have not obtained the market expansion standards or cost savings benefits (Molla and Heeks, 2007). Especially for small and medium-sized enterprises, the complexity of cross-border transactions and the lack of their own resources determine that it is difficult to obtain the benefits of cross-border trade (Patterson and Wilson, 2000). In addition, the increase in transaction uncertainty caused by the lack of information further weakens the perceived benefits of cross-border e-commerce sellers.

We argue that social support positively affects the perceived benefits of cross-border e-commerce sellers. First of all, valuable information provided by group members, especially members similar to the sellers themselves, can reduce the cost of searching information (Xiao et al., 2015). Second, more social support means more sources of information. The more information the seller contacts, the more the seller will understand the latest developments in the product market, and thus more accurately grasp the needs of consumers. This helps increase the company’s sales performance. Finally, while cross-border transactions bring benefits to sellers, they are also accompanied by transaction risks, especially for cross-border e-commerce. The existence of risks will inhibit people’s perceived benefits (Chaudhuri, 1998). Thus, a large amount of relevant information can effectively weaken people’s perception of the risks of transactions (Zhang et al., 2015), which will promote sellers’ perceived benefits. Compared with information support, the existence of emotional support mainly indirectly helps people overcome difficulties with a positive attitude. This positive attitude will inhibit individuals’ perception of risks, thereby increasing their perceived benefits. Therefore, we put forward the following hypothesis.

H2a: Information support is positively associated with perceived benefits.
H2b: Emotional support is positively associated with perceived benefits.

### The Mediating Role of Perceived Benefits

Zhang et al. (2015) pointed out that the perceived benefits will mediate the relationship between the knowledge that consumers have and their purchase intention. A large amount of relevant knowledge can help consumers obtain more useful information. As a result, the perceived risk to consumers will be reduced and the perceived benefits will increase accordingly, which will increase consumers’ willingness to buy (Zhang et al., 2015). Based on this, this study believes that perceived benefit is an important intermediary variable between the seller’s perceived social support and the willingness to retain. The foundation of a business is profitability. When sellers feel that cross-border e-commerce is profitable, they will have a stronger willingness to retain. Sellers are more aware of the benefits of cross-border e-commerce. One of the reasons sellers are able to perceive more benefits of cross-border e-commerce is that the valuable information provided by others can weaken the perception of risk, and some advisory information can directly help sellers overcome difficulties and make some decision correctly (Liang and Turban, 2011). In addition, emotional support from others, such as care, understanding and respect, allows sellers to actively seek solutions when facing difficulties, indirectly helping them to solve the problems they encounter (Pfeil and Zaphiris, 2009). Finally, in the cross-border e-commerce industry, when sellers gain the understanding and care from people who have similar painful experiences, it will make them release and anchor their emotions and feel comfortable and belonging, which will further motivate them to overcome difficulties and retain. Therefore, the following hypotheses are proposed in this paper.

H3: Perceived benefits is positively correlated with sellers’ willingness to retain.
H4a: Perceived benefits mediates the relationship between information support and sellers’ willingness to retain.
H4b: Perceived benefits mediate the relationship between emotional support and sellers’ willingness to retain.

### Moderating Effect of Perceived Usefulness

The concept of perceived usefulness comes from the technology acceptance model, which refers to an individual’s subjective perception of whether the use of new technology can help improve work efficiency. Specifically, it refers to the user’s belief or expectation that the use of a system/IT will improve his efficiency or results in accomplishing a task (Venkatesh et al., 2003). For the sellers in this study, the perceived usefulness refers to the evaluation of whether the use of third-party platforms can improve efficiency and operating performance of cross-border transaction. Some studies have pointed out that perceived usefulness will affect consumers’ purchase intention. For example, Moslehpour et al. (2018) propose that perceived usefulness is related to consumers’ purchase intention. Yoon and Steege (2013), Hajli (2014b), and Bonn et al. (2016) also confirmed this point. Different from previous studies, our research object is the perceived usefulness of third-party platforms of cross-border e-commerce sellers. Through small-scale interviews, we believe that sellers’ perceived usefulness of the platform will influence the relationship between perceived benefits and willingness to retain. Because the completion of the seller’s cross-border transaction needs to rely on the support of the platform, different people have different perceptions of the usefulness of the platform. In other words, people will have different perceived usefulness to the same platform. For example, in the case study of Charpin et al. (2021), restaurants have significantly different evaluations of the usefulness of mobile procurement platforms. The usefulness of the platform is critical to the availability of sellers to turn the perceived benefits into real benefits. Therefore, we believe that the level of perceived usefulness will have an interacting effect with the perceived benefits upon the sellers’ willingness to retain. Specifically, when an individual perceives a higher platform usefulness, he believes that the platform he relies on can turn the perceived benefits into the actual benefits of the enterprise and increase his confidence in cross-border e-commerce transactions. This will further strengthen the relationship between perceived benefits and sellers’ willingness to retain. Conversely, the impact of perceived revenue on sellers’ willingness to retain will be weakened.

In addition, this study believes that perceived usefulness not only moderates the relationship between perceived benefits and sellers’ willingness to retain, but also moderates the conditional indirect relationship between social support and sellers’ willingness to retain (transmitted by perceived benefits). As mentioned earlier, the perceived benefit act as the mediating variables between information support and emotional support on sellers’ willingness to retain. And in the case of high perceived usefulness, the seller’s retention will be further enhanced by the impact of perceived benefits. Based on this, this study argues that in the case of high perceived usefulness, information support and emotional support have a stronger indirect effect on sellers’ retention intentions through perceived benefits. Therefore, the following hypothesis is proposed.

H5: Perceived usefulness moderates the influence of perceived benefits on sellers’ willingness to retain, such that the effect is stronger with the high perceived usefulness.
H6a: Perceived usefulness moderates the indirect effect of information support on sellers’ willingness to retain, such that the effect is stronger with the high perceived usefulness.
H6b: Perceived usefulness moderates the indirect effect of emotional support on sellers’ willingness to retain, such that the effect is stronger with the high perceived usefulness.

## Research Design

### Scale Description

In order to obtain a sample that meets the requirements, the first part of the questionnaire sets up three selection translations: (1) The province where the individual is located. When the respondent chooses “Fujian,” the survey will continue, otherwise the survey will end; (2) The personal residential address, when the respondent chooses “Quanzhou,” the survey will continue, otherwise the survey will end; (3) Individuals are engaged in the industry. The investigation will continue when the subject chooses the “cross-border e-commerce industry,” otherwise the investigation will end. In order to improve the effectiveness of data sources, each person participating in the survey will be paid. The second part of the questionnaire is a survey on retention expectations of cross-border e-commerce sellers. The factors that affect the willingness of cross-border e-commerce sellers to retain include social support, perceived benefits, and perceived platform usefulness. Each variable has 3–10 questions, and there are 22 questions in total. In order to prevent demographic variables from affecting the research conclusions, gender, age, and education are set as control variables, and other variables are measured using the Likert five-point scale. The questions of the questionnaire were all selected from the maturity scales in the existing literature, followed by small-scale interviews and discussions. According to the interviewer’s suggestion, the questionnaire was revised to make it more suitable for the survey situation of this study. Finally, the measurement items of each variable are shown in Table 2.

**TABLE 2:** Scales of the constructs.

Construct	Items		Resources
Social support	Information support	When I encounter difficulties, some of the cross-border e-commerce practitioners who interact with me can provide me with some information and help me solve the difficulties.	Liang et al., 2011; Hajli, 2014a
		When I need help, some of the cross-border e-commerce practitioners who interact with me can give me advice.	
		When I face difficulties, some of the cross-border e-commerce practitioners who interact with me can help me find the cause of the problem and provide suggestions.	
	Emotional support	When I faced difficulties, some of the cross-border e-commerce practitioners who interacted with me are on my side with me.	
		When I face difficulties, some of the cross-border e-commerce practitioners who interact with me will comfort and encourage me.	
		When I face difficulties, some of the cross-border e-commerce practitioners who interact with me will expressed interest and concern in my well-being.	
Perceived benefits	Interaction with other cross-border e-commerce practitioners can help me reduce operation costs (personnel, rent, order, and payment processing).		Molla and Heeks, 2007
	Interaction with other cross-border e-commerce practitioners can help me reduce market costs (communications, interaction, customer information management, bypassing intermediaries).		
	Interaction with other cross-border e-commerce practitioners can help me reduce the cost of maintaining up-to-date company information.		
	Interaction with other cross-border e-commerce practitioners can help me reduce the company’s transaction costs (purchasing, sales).		
	Interaction with other cross-border e-commerce practitioners can help me extending firm’s reach (market).		
	Interaction with other cross-border e-commerce practitioners can help me improve the differentiation of products and services.		
	Interaction with other cross-border e-commerce practitioners can help me improve competitive position.		
	Interaction with other cross-border e-commerce practitioners can help me to promote communication between companies.		
	Interaction with other cross-border e-commerce practitioners can help me increase revenue.		
	Interaction with other cross-border e-commerce practitioners can help me improve the company’s image.		
Perceived usefulness	This platform can improve my performance in product search and product sales.		Davis, 1989
	This platform allows me to be more efficient in product search and product sales.		
	This platform can enhance my effectiveness in product search and product sales.		
Seller’s willingness to retain	Given the chance, I predict that I would consider continuing to engage in cross-border e-commerce transactions in the future.		Liang et al., 2011; Guo et al., 2018
	It is likely that I will continue to work in cross-border e-commerce in the future.		
	Given the opportunity, I intend to sell products to cross-border consumers.		

### Data Collection and Samples

This article mainly investigates the sellers of cross-border e-commerce. Quanzhou is the origin of the “Maritime Silk Road,” and cross-border transactions have deep historical and cultural heritage here. In the “Report on the Development Trends of China’s Export E-commerce Cities with Amazon Global Store Opening” released in 2018, Quanzhou was ranked among the “Top 20 Chinese Export E-commerce Cities Development with Amazon Global Stores.” According to incomplete statistics, there are currently more than 4,000 cross-border e-commerce sellers in Quanzhou, of which about 1,000 are export cross-border e-commerce B2C companies. Therefore, this article selects some cross-border e-commerce sellers in Quanzhou as the survey objects, which is representative.

The descriptive statistical results of the sample are as follows: Among the cross-border e-commerce sellers surveyed, in terms of gender, men accounted for 70.44% and women accounted for 29.56%; in terms of age, those aged 25 and below accounted for 10.34%, those aged 26–35 accounted for 67.00%, those aged 36–45 accounted for 19.21%, and those aged 46 and above accounted for 3.45%; in terms of education level, junior high school and below accounted for 7.39%, high school accounted for 10.84%, technical secondary school accounted for 14.78%, and junior college accounted for 27.09%, bachelor degree accounted for 36.45%, master degree and above accounted for 3.45%.

## Results

### Measurement Model

The reliability and validity of the variables and the research model of this study were analyzed using SmartPLS 3. PLS, a PLS structural equation modeling tool. This tool has notable advantages, such as minimal demands on sample size, sample distribution and measurement scales. It also excels at the simultaneous analysis in which the conceptual model is quite complex (Hair et al., 2014).

Table 3 shows the Cronbach’s α, composite reliability (CR), and average variance extracted (AVE) values of each variable. It can be seen from the table that, the values of Cronbach’s α of each variable are greater than the threshold 0.7. This shows that the questions of the scale have high credibility. The values of CR are greater than 0.7, indicating that the scale has good combination reliability. The values of AVE for all constructs are greater than 0.5, indicating that the scale has good convergent validity. Thus, the measurement items appear reliable and converged on the latent constructs.

**TABLE 3:** Reliability and convergent validity.

Construct	Cronbach’s α	CR	AVE
Emotional support	0.770	0.866	0.684
Information support	0.722	0.844	0.645
Perceived benefits	0.928	0.940	0.609
Perceived usefulness	0.805	0.884	0.719
Seller’s willingness to retain	0.848	0.908	0.767

*CR, composite reliability; AVE, average variance extracted.*

To test the discriminant validity, we followed the guidelines of Fornell and Larcker (1981) and Chin (1998). As shown in Table 4, the correlation coefficient between the variables is less than 0.8, and the square roots of the AVEs are greater than the correlation coefficient between all variables, so the discrimination validity is passed (Fornell and Larcker, 1981). As shown in Table 5, the factor loadings of all items on their respective construct are range from 0.7 to 0.95 (*P* < 0.0001); and more strongly on their respective construct than on any other. Thus, the discrimination validity of the constructs in our research model is supported.

**TABLE 4:** Analysis of discriminative validity.

	ES	IS	PB	PU	SWTR	Age	Education	Gender
ES	**0.827**							
IS	0.575	**0.803**						
PB	0.772	0.599	**0.781**					
PU	0.757	0.502	0.742	**0.848**				
SWTR	0.608	0.588	0.688	0.675	**0.876**			
Age	0.022	0.258	0.108	0.065	0.108	**NA**		
Education	–0.326	0.013	–0.197	–0.311	–0.199	0.369	**NA**	
Gender	–0.078	–0.008	–0.076	–0.047	–0.016	–0.008	0.231	**NA**

*The bold font on the diagonal are the square roots of the AVEs and the lower triangle is the Pearson correlation coefficient.*

**TABLE 5:** Factor loadings (bolded) and cross-loadings.

	ES	IS	PB	PU	SWTR
ES1	**0.818**	0.590	0.580	0.607	0.503
ES2	**0.785**	0.411	0.550	0.601	0.432
ES3	**0.876**	0.436	0.759	0.667	0.560
IS1	0.350	**0.705**	0.400	0.300	0.385
IS2	0.477	**0.875**	0.528	0.443	0.533
IS3	0.543	**0.819**	0.504	0.450	0.485
PB1	0.689	0.376	**0.773**	0.611	0.532
PB2	0.646	0.645	**0.809**	0.612	0.600
PB3	0.619	0.514	**0.863**	0.594	0.669
PB4	0.624	0.619	**0.830**	0.586	0.604
PB5	0.503	0.526	**0.719**	0.508	0.498
PB6	0.564	0.387	**0.737**	0.515	0.375
PB7	0.672	0.374	**0.764**	0.630	0.464
PB8	0.641	0.312	**0.714**	0.596	0.461
PB9	0.579	0.418	**0.817**	0.594	0.520
PB10	0.479	0.452	**0.767**	0.538	0.590
PU1	0.650	0.238	0.520	**0.831**	0.449
PU2	0.640	0.494	0.674	**0.798**	0.546
PU3	0.646	0.500	0.673	**0.910**	0.682
SWTR1	0.620	0.505	0.622	0.676	**0.901**
SWTR2	0.438	0.476	0.559	0.519	**0.851**
SWTR3	0.527	0.561	0.622	0.569	**0.875**

### Hypothesis Testing

**FIGURE 2:**
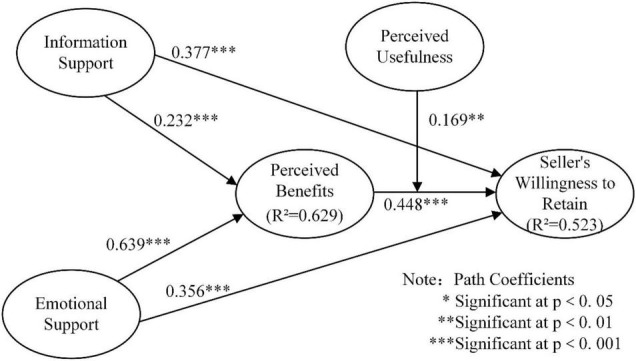
Structural model results.

Figure 2 shows the results of empirical test of the structural model, all hypotheses received strong support. It can be seen from Figure 2 that information support had a positively influence on sellers’ willingness to retain (β = 0.377, *t* = 4.735, *P* < 0.001), and emotional had a positively influence on sellers’ willingness to retain (β = 0.0.356, *t* = 3.984, *P* < 0.001), H1a and H1b are supported. Information support had a positive impact on perceived benefits (β = 0.232, *t* = 3.542, *P* < 0.001), emotional support had a positive impact on perceived benefits (β = 0.639, *t* = 12.135, *P* < 0.001), H2a and H2b have been verified. Perceived benefits had a positively influence on seller’s willingness to retain (β = 0.448, *t* = 4.144, *P* < 0.001), H3 is supported.

In order to test the mediation effect suggested in H4a and H4b in Figure 1, we computed t-statistics and path significance levels for each of the hypothesized relationships using the bootstrapping method. The test results are shown in Table 6, and the results confirm that the perceived benefits mediate the influence of information support (β = 0.104, *t* = 2.439, *P* < 0.050) and emotional support (β = 0.286, *t* = 3.995, *P* < 0.001) on sellers’ willingness to retain. Thus, H4a and H4b are supported.

**TABLE 6:** Summary of the tests of mediating effects.

Dependent variable Seller’s willingness to retain	Results			Bootstrap results			
				Point estimate	*p*-value	Boot LL 97.5%CI	Boot UL 97.5%CI
Independent variable	Mediating variable	*t*-value	Mediation				
Emotional support	Perceived benefits	3.995	Yes	0.286	0.000	0.147	0.429
Information support	Perceived benefits	2.439	Yes	0.104	0.015	0.037	0.204

To test the interaction effects, we formulated the interaction term by multiplying the corresponding indicators of the predictor and moderator constructs. As shown in Figure 2, statistically significant beta path coefficient was indicated, perceived usefulness had a positive interacting effect with perceived benefits on sellers’ willingness to retain (β = 0.169, *t* = 2.685, *P* < 0.010). Thus, H5 is supported.

In order to test the moderated mediation effect suggested in H6a and H6b in Figure 1, consistent with the guidelines of Preacher et al. (2007), we used the bootstrap procedures (Hayes PROCESS, select Model 14) implemented in Preacher et al.’s application in these analyses. Model 14 includes formal significance tests of the indirect effect between the independent and the dependent variable, as transmitted by the mediating variable, at different values of the moderator (Menges et al., 2011). In other words, we evaluated the statistical significance of the conditional indirect relationship between our measures of social support and seller’s willingness to retain, as transmitted by perceived benefits, at the mean value of perceived usefulness and at one standard deviation below and above the mean.

The results of the moderated mediation analyses are shown in Table 7. The conditional indirect relationship between information support and emotional support and sellers’ willingness to retain (transmitted by perceived benefits) are moderated by perceived usefulness. Specifically, the conditional indirect relationship of information support with seller’s willingness to retain (through perceived benefits) does not reach the statistical significance at the mean value (boot indirect effect = 0.011; ns) or at one standard deviation below the mean of perceived usefulness (boot indirect effect = 0.054; ns). However, the conditional indirect relation is positive and statistically significant, at one standard deviation above the mean value of perceived usefulness (boot indirect effect = 0.097; sig), it means that the conditional indirect relation is positive and statistically significant, H6a is supported. Similarly, the conditional indirect relationship of emotional support with seller’s willingness to retain (transmitted by perceived benefits) does not reach the statistical significance at the mean value (boot indirect effect = 0.033; ns) or at one standard deviation below the mean of perceived usefulness (boot indirect effect = 0.116; ns). When the value of perceived usefulness is higher than the mean value by one standard deviation (boot indirect effect = 0.200; sig), meaning the conditional indirect relation is positive and statistically significant, H6b is supported.

**TABLE 7:** Moderated mediation model test.

Perceived benefits	Emotional support				Information support			
	Boot indirect effect	Boot SE	Boot LL 95%CI	Boot UL 95%CI	Boot indirect effect	Boot SE	Boot LL 95%CI	Boot UL 95%CI
–1 SD Mean +1 SD	0.033 0.116 0.200	0.074 0.068 0.081	–0.107 –0.017 0.048	0.178 0.255 0.371	0.011 0.054 0.097	0.042 0.042 0.054	–0.074 –0.014 0.018	0.093 0.151 0.236

## Discussion

### Conclusion

This paper takes cross-border e-commerce sellers as the research object and proposes a structural model that affects cross-border e-commerce sellers’ retention willingness based on the S-O-R theory. The findings of our study are as follows: firstly, both information support and emotional support have positive influence on sellers’ willingness to retain. This corresponds to previous research showing that the social support had an influence on consumers’ behavioral intentions (Zhu et al., 2016; Lin et al., 2018; Wang et al., 2020). Compared with domestic transactions, cross-border transactions not only bring a larger market for sellers, but also greater transaction risks and uncertainties. One of the reasons is the asymmetry of information between buyers and sellers. Through communication and information sharing between sellers, it will increase the understanding of consumers and increase the probability of successful transactions. In addition, mutual encouragement and support among sellers will bring a strong sense of belonging. All in all, both the benefits of a successful transaction and the support from others when faced with difficulties will increase sellers’ willingness to continue to engage in cross-border e-commerce.

Secondly, perceived benefits of sellers had a positive influence on sellers’ willingness to retain. Specifically, perceived benefits are feelings in the mind of sellers, reflecting in the sellers’ awareness of the benefits of engaging in cross-border transactions. Such awareness reflects the seller’s attitude toward cross-border transaction activities. When a seller feels that cross-border transactions can bring greater benefits, he is more likely to continue to participate in cross-border transactions. Conversely, sellers are more likely to choose domestic transactions, which are less risky.

Thirdly, perceived benefits act as a mediating variable between the two antecedents (information support, emotional support) and sellers’ willingness to retain. A large amount of valuable information can help sellers reduce the uncertainty of transactions, so that the seller’s perceived risks will be reduced and the perceived benefits will increase accordingly. The pursuit of profit is fundamental to the businessman. Only when the seller believes that cross-border transactions are profitable can he continue to engage in cross-border e-commerce transactions instead of turning to other industries.

Finally, perceived usefulness had an interacting effect with perceived benefits on seller’s retention intention. The conditional indirect relationship between information support and emotional support and sellers’ willingness to retain (transmitted by perceived benefits) are moderated by perceived usefulness. Perceived benefit is a kind of seller’s cognition, and whether this cognitive benefit can be turned into real benefit requires the help of a third-party platform. When the seller thinks that the third-party platform can’t help them translate perceived benefits into real benefits, the seller’s perception of benefits is only at the cognitive level and cannot be put into practice. Therefore, the impact of perceived benefits on willingness to retain will be reduced. Only when sellers perceive that the use of third-party platforms can bring positive feedback to cross-border e-commerce business, the perceived benefits brought by social support can be worked. In addition, we believe that perceived usefulness plays a crucial role in influencing the relationship between social support, perceived benefits, and willingness to retain. Only if sellers perceive the higher usefulness of third-party platforms, social support will indirectly affect the retention willingness of cross-border e-commerce sellers through perceived benefits. Because in the case of low perceived usefulness of the platform, sellers may doubt the value of the information they receive or the authenticity of the information, thereby reducing the seller’s perceived benefits and further reducing the willingness to retain. Conversely, when the perceived usefulness of the platform is high, sellers will be more convinced of the reliability and value of social support, thereby increasing their perceived benefits and increasing their willingness to retain.

### Theoretical Contributions

In the context of cross-border e-commerce, sellers play an equally important role like buyers. Without buyers, sellers will not be able to sell their products. Similarly, without sellers, it will be difficult for buyers to buy their favorite products. Therefore, cross-border e-commerce sellers and buyers are two important roles in cross-border e-commerce transactions. However, most of previous literature about cross-border e-commerce focused on the benefits of buyers and discusses how to improve buyers’ willingness to buy. They assume that the buyer is the weaker party in cross-border transactions (Hong, 2015; Kim and Koo, 2016) and only consider the impact of the seller’s opportunistic behavior on the buyer. Different from previous research, this paper calls attention to increasing sellers’ willingness to retain based on the complex facts of cross-border e-commerce, such as chargeback fraud, e-commerce platforms’ preference for buyers’ rights, sellers’ “difficulty to retention” and so on. Specifically, based on the S-O-R theory, this paper develops a conceptual model to examine the antecedents of sellers’ perceived benefits and their influence on sellers’ willingness to retain. In addition, considering the important role played by third-party platforms in cross-border e-commerce transactions, this paper also examines the impact of perceived benefits and its antecedents on sellers’ willingness to retain under different levels of platform perceived usefulness. In so doing, this paper has the following three main contributions.

Firstly, this study extends the existing literature on the determinants and consequences of perceived benefits from the perspective of the seller. Most of the previous studies focused on the antecedents and consequences of buyers’ perceived benefits from the perspective of consumers (Chae et al., 2020; Li et al., 2020; Lin et al., 2020; Sinha and Verma, 2020). Although these studies have increased our understanding of cross-border e-commerce buyers, the success of e-commerce requires not only buyers, but also sellers (Sun, 2010). From a new perspective of cross-border e-commerce sellers, based on this, this paper proposed and tested the influence of different dimensions of social support on the sellers’ willingness to retain through their perceived benefits. We have found that information support and emotional support can help increase sellers’ perceived benefits, because a large amount of information support can help sellers obtain more valuable information, reduce transaction uncertainty, and increase the probability of transaction success. Different from information support, more emotional support can help sellers face the possible risks of cross-border trading centers with a positive attitude and increase their perceived benefits. To sum up, all of these are important factors affecting sellers’ willingness to retain. Therefore, the findings of this paper enrich the related research on the antecedents and consequences of perceived benefits. For example, existing research focused on the antecedents and consequences that affect perceived benefits of consumers (Chae et al., 2020; Li et al., 2020; Lin et al., 2020; Sinha and Verma, 2020). However, this paper explores the antecedents and consequences of perceived benefits of cross-border e-commerce sellers.

Secondly, the findings of this paper show that information support and emotional support obtained by the sellers will affect the seller’s willingness to retain through their perceived benefits. This finding emphasizes the importance of paying attention to the seller’s rights and interests, which is in sharp contrast to the mainstream view that only paying attention to the buyer’s rights and interests in the existing research. Specifically, the existing research believes that the development of cross-border e-commerce mainly depends on the buyer’s willingness to buy continuously, and it is assumed that buyers are more easily influenced by the harmful effects of sellers’ opportunistic behavior (Guo et al., 2018). Therefore, most of the existing studies only start from the buyer’s perspective, discussing how to protect consumers’ rights (Wang et al., 2021) and increase consumers’ purchase intentions (Escandon-Barbosa and Rialp-Criado, 2019; Liu et al., 2020; Guitart and Stremersch, 2021). This research shows that, due to the complexity of cross-border e-commerce transactions and the impact of information asymmetry, sellers are also vulnerable to opportunistic behaviors by buyers, such as chargeback fraud. This will seriously damage the benefits of sellers and their willingness to continue to engage in cross-border e-commerce. Therefore, in the situation of cross-border e-commerce, exploring to improve the seller’s willingness to retain is just as important as paying attention to the interests of buyers. This paper meets the lack of attention paid to sellers’ rights gap by shifting the focus to the seller’s perceived benefits and retention willingness. This paper is a natural extension of related research in e-commerce research on improving buyers’ purchasing intention (Wei et al., 2018; Lin et al., 2019; Chen and Yang, 2021; Kowalczuk et al., 2021). By exploring how to improve sellers’ willingness to retain, in this sense, this paper contributes to the promotion of research on sellers’ behavior in the cross-border e-commerce and enriches the existing cross-border e-commerce research.

Finally, this paper considers the perceived usefulness of cross-border e-commerce sellers to the third-party trading platform they rely on and finds that the perceived usefulness of sellers to the platform influences the relationship between sellers’ perceived benefits and retention intention. This finding emphasizes the importance of third-party trading platforms to provide rights and interests protection for sellers in cross-border transactions. Specifically, cross-border transactions are mostly conducted on third-party trading platform. The research shows that the perceived usefulness of third-party trading platforms plays an important role in e-commerce transactions (Lee et al., 2019; Abbes et al., 2020; Kwak et al., 2020). However, existing studies mainly focus on the impact of platform perceived usefulness on buyers’ continuous use of the website and purchase intention, with few studies explore the impact of perceived usefulness on sellers’ willingness to retain. In addition, the excessive protection of consumers’ rights and interests by third-party platforms, such as some online platforms that do not allow sellers to leave negative comments on buyers (Sun, 2010), which encourages buyers’ opportunistic behavior and increases the risk of damage to sellers’ interests. This paper finds that the higher the perceived usefulness of seller to the trading platform, the stronger the sellers believes that the platform protects their interests. It means that the platform they rely on is more likely to translate the perceived benefits into the actual income of the enterprise, thereby enhance the impact of the seller’s perceived benefits on the seller’s retention intention. In general, these findings emphasize the importance of sellers’ social support and perceived benefits, and the necessity of third-party platforms to protect sellers’ legitimate interests when designing cross-border online transaction mechanisms. In this sense, through the research on how to protect the interests of sellers and improve sellers’ retention intention, this paper not only provides an important supplement to the mainstream view on the necessity of protecting buyers from sellers’ opportunistic behavior in the existing literature (Kim and Koo, 2016; Meents and Verhagen, 2018), but also provides a new idea for the future research related to the impact of perceived usefulness.

### Practical Contributions

This study provided new insights and solutions for promoting the sustainable development of cross-border e-commerce. Firstly, our findings affirm the crucial role of social support in promoting sellers’ willingness to retain. Thus, for cross-border e-commerce sellers themselves, we suggest they must manage their own social relationships in the industry, manage the relationship of competition and cooperation with other sellers, and expand sources of information and emotional support, because social support can help them reduce the risks and costs in cross-border transactions.

Secondly, we found that sellers’ perceived usefulness of third-party platforms is also an important factor affecting sellers’ willingness to retain. As mentioned earlier, the development of cross-border e-commerce depends not only on buyers, but also on sellers. Therefore, in order to improve sellers’ willingness to retain, the third-party trading platforms must understand that sellers and buyers are equally important to the platform. And we advise the third-party trading platform should improve the transaction mechanism to avoid unnecessary losses for the seller’s legitimate interests, only in this way can the third-party platform itself and the entire cross-border e-commerce industry develop well.

Finally, to create a mutually beneficial and healthy industry atmosphere, the policy makers of cross-border e-commerce industry associations in various regions should actively encourage sellers to exchange their experience, help each other, exchange what they have and learn from each other, which will help sellers get more benefits from cross-border transactions.

### Limitations and Future Research

Finally, this study also has some limitations. First, self-questionnaire survey is used in data collection, and there may be some methodological bias. Second, the questionnaire survey is conducted through the Internet, although the respondents are given some reward, it is still unable to guarantee that all the answers are true and objective. Finally, the respondents are cross-border e-commerce sellers in Quanzhou, and the research results may not be applicable to other regions. In future research, researchers can consider expanding the source of respondents, and at the same time, we can use online and offline data collection to test the universality and effectiveness of the model.

## Data Availability Statement

The raw data supporting the conclusions of this article will be made available by the authors, without undue reservation.

## Author Contributions

HS and XZ contributed to conception and design of the study. JY and HZ organized the database. JY and JG performed the statistical analysis. HS wrote the first draft of the manuscript. XZ wrote sections of the manuscript. All the authors contributed to manuscript revision, read, and approved the submitted version.

## Conflict of Interest

The authors declare that the research was conducted in the absence of any commercial or financial relationships that could be construed as a potential conflict of interest.

## Publisher’s Note

All claims expressed in this article are solely those of the authors and do not necessarily represent those of their affiliated organizations, or those of the publisher, the editors and the reviewers. Any product that may be evaluated in this article, or claim that may be made by its manufacturer, is not guaranteed or endorsed by the publisher.
